# Risk of depression in multiple sclerosis across disease-modifying therapies

**DOI:** 10.1177/13524585211031128

**Published:** 2021-07-15

**Authors:** Elisa Longinetti, Thomas Frisell, Simon Englund, Johan Reutfors, Fang Fang, Fredrik Piehl

**Affiliations:** Department of Clinical Neuroscience, Karolinska Institutet, Stockholm, Sweden; Department of Medicine Solna, Clinical Epidemiology Division, Karolinska Institutet, Stockholm, Sweden; Department of Clinical Neuroscience, Karolinska Institutet, Stockholm, Sweden; Department of Medicine Solna, Clinical Epidemiology Division, Karolinska Institutet, Stockholm, Sweden; Institute of Environmental Medicine, Karolinska Institutet, Stockholm, Sweden; Department of Clinical Neuroscience, Karolinska Institutet, Stockholm, Sweden

**Keywords:** Multiple sclerosis, depression, antidepressants, cohort studies, disease-modifying therapies, rituximab

## Abstract

**Background:**

Depression and use of antidepressants are more common among patients with multiple sclerosis (MS) compared to the general population, but the relation of psychiatric comorbidity to use of different disease-modifying therapies (DMTs) is less clear.

**Objective:**

To determine whether risk of incident depression or antidepressant use differed across DMTs, and to assess whether depression and antidepressants affected risk of DMT discontinuation and MS relapses.

**Methods:**

We prospectively followed for 8 years a register-based nationwide cohort of 3803 relapsing-remitting MS patients.

**Results:**

Patients on rituximab had a lower risk of being diagnosed with depression or initiating antidepressants compared with the reference group treated with interferons (hazard ratio (HR) = 0.72, 95% confidence interval (CI) = 0.54–0.96). Patients diagnosed with depression discontinued interferon treatment to a higher extent than patients without depression (HR = 1.51; 95% CI = 1.15–1.98), as did patients on fingolimod initiating an antidepressant compared to patients who did not initiate an antidepressant (HR = 1.47; 95% CI = 1.04–2.08).

**Conclusions:**

Our results indicate that the choice of DMT is associated with subsequent risk of depression in MS, but further studies are needed to establish whether there is a causal link. Overall, depression and use of antidepressants displayed limited associations with DMT discontinuation and MS relapse.

## Introduction

Depression is a frequent comorbidity in multiple sclerosis (MS) and considerably reduces patients’ quality of life,^1^ regardless of disability status.^2^ Clinically significant depressive symptoms occur in 19%–40% of MS patients, double the reported prevalence in the general population.^1^

The etiology of depression in MS is likely multifactorial and may include both psychological and biological components.^1^ The stress reaction of receiving an MS diagnosis may contribute to the elevated risk of depression diagnosis and antidepressant use.^3^ Other evidence suggests an association between depression and risk of subsequent MS diagnosis, not attributable to genetic liability.^4^ Some elements of neuroinflammation may also represent biological denominators for both conditions.^5,6^

Whether disease-modifying therapies (DMTs) modify the risk of depression in relapsing-remitting MS (RRMS) is unclear, and comparisons across newer DMTs are rare. The main objective of this study was to determine whether DMT choice modulated the subsequent risk of being diagnosed with depression or using antidepressants in a large nationwide cohort of RRMS patients with no history of depression or antidepressant use before first DMT. The secondary aim was to examine whether occurrence of depression or use of antidepressants affected the risk of DMT discontinuation or experiencing an MS relapse.

## Method

We performed a nationwide cohort study of RRMS patients, free of diagnosed depression and antidepressant use prior to first DMT, linking the Swedish MS Registry to national healthcare and census registers.

### Study design

The Swedish MS Registry is a web-based resource collecting high-quality healthcare data for all MS patients in Sweden since 2001.^7^ It is an integrated part of Swedish MS care; patients are informed orally about the data collection process and are free to opt out.^7^ Despite participation in the registry being voluntary, both for the patients and their attending neurologists, the registry includes about 80% of MS patients in Sweden with approximately 18,000 patients in total.^7^ Consistent with national guidelines recommending treatment for MS patients with active disease,^2^ only about 5% of patients have no recorded DMT in the MS Registry (including patients with missing therapy data). Overall, data collected in this registry have been shown as both accurate and complete.^8^ We defined our study population as all patients included in the Swedish MS Registry residing in Sweden from 5 years prior to MS diagnosis or longer and first diagnosed with RRMS from January 2010 to September 2018 (*N* = 4771). Through the unique personal identification numbers assigned to all Swedish residents,^9^ we cross-linked our study population to the Swedish Migration Register^10^ and the Swedish Causes of Death Register,^11^ to follow the cohort from DMT start until emigration out of Sweden, death, withdrawal from the Swedish MS Registry, or end of follow-up (31 December 2018), whichever came first.

### Ascertainment of depression diagnosis and antidepressant use

We further cross-linked our study population to the Swedish Patient Register^12^ and the Swedish Prescribed Drug Register^13^ to extract information about depression diagnosis and antidepressant use. The Swedish Patient Register collects information about inpatient care since 1964, and hospital-based outpatient specialist care since 2001. Each diagnosis in the Patient Register is classified according to the Swedish Revisions of the International Classification of Diseases (ICD) codes (ICD-10 from 1997). The Prescribed Drug Register includes information on all dispensed prescribed drugs in Sweden since July 2005, classified according to the Anatomical Therapeutics Chemical (ATC) system. We identified clinical diagnosis of depression at inpatient or outpatient visits, using ICD-10 codes F32-34 or F38-39 (*N* = 519, 10.9%). We defined patients as antidepressant users if they had filled at least two antidepressant prescriptions, with the date of first prescription fill used as the start date. We identified antidepressant prescription fills, including selective serotonin reuptake inhibitors (SSRIs), non-selective monoamine oxidase (MAO) inhibitors, MAO A inhibitors, and other antidepressants (including serotonin and norepinephrine reuptake inhibitors, but not duloxetine) using ATC codes N06AB, N06AF, N06AG, and N06AX except N06AX21 (*N* = 1370, 28.7%). Non-selective monoamine reuptake inhibitors including tricyclic antidepressants (ATC code N06AA) and duloxetine (ATC code N06AX21) were excluded because they may be used to treat neuropathic pain in MS patients^14^ (*N* = 582, 12.2%). We excluded RRMS patients who did not have any recorded DMT (*N* = 245, 5.1%) and patients who were diagnosed with depression or had filled an antidepressant prescription during the 5 years prior to the start of first DMT (*N* = 723, 5.72%), leaving 3803 patients in the analyses (79.7%).

### Depression and antidepressant use in relation to different DMTs

Because patients could have different DMTs during the follow-up, we considered DMTs as time-varying variables and compared all time periods when patients were treated with dimethyl fumarate, fingolimod, glatiramer acetate, natalizumab, or rituximab, with time periods when patients were treated with interferons (interferon beta-1a and interferon beta-1b). Other DMTs were used less frequently, and collapsed to one group including alemtuzumab, daclizumab, hematopoietic stem cell transplantation, mitoxantrone, and teriflunomide. We followed all patients from start of each DMT to date of depression diagnosis or antidepressant prescription filling, DMT discontinuation, migration out of Sweden, withdrawal from the Swedish MS Registry, or end of follow-up. Patients who switched DMTs contributed to multiple DMT cohorts.

### DMT discontinuation or MS relapse in relation to depression or antidepressant use

We regarded depression diagnosis and antidepressant use as time-varying exposures and considered patients exposed from the date of first depression diagnosis or first prescription fill of antidepressants. We followed all patients from start of DMT to date of DMT discontinuation or relapse, migration out of Sweden, withdrawal from the MS Registry, or end of follow-up. Since DMT discontinuation was interpreted as a poor treatment outcome, the following were considered censoring events and not counted as outcome events: discontinuation with the stated reason being a stable condition, planned pregnancy, or pregnancy.

### Potential confounding factors

Potential confounders included demographic factors, psychiatric comorbidity, and MS severity. Demographic factors included sex, country of birth, education, age at DMT start, and geographical region of treatment. We extracted information about education from the Swedish Longitudinal Integrated Database for Health Insurance and Labor Market Studies^15^ and selected for each observation the highest achieved education level at DMT start. We defined history of (1) bipolar disorder, (2) anxiety disorders, and (3) all other mental and behavioral disorders by the diagnoses of these disorders in the Swedish Patient Register during the 5 years before first DMT start (bipolar disorder ICD-10 codes: F30-F31, anxiety disorders: F40-48, all other mental and behavioral disorders: F00-F99 except F30-34, F40-48, or F38-39). We assessed MS severity through disease duration, that is., time since MS diagnosis at DMT start, DMT line, that is., number of previous DMTs, the Expanded Disability Status Scale (EDSS), and physical and psychological components of the MS Impact Scale (MSIS-29), recorded in the Swedish MS Registry. For each observation we selected the closest measurement recorded prior to DMT start. In the analysis of DMT discontinuation or relapse in relation to depression or antidepressant use we selected the closest measurement recorded prior to DMT start and prior to depression diagnosis or antidepressant prescription fill, if the patient was exposed to depression or antidepressant use.

### Statistical analyses

We derived hazard ratios (HRs) and the corresponding 95% confidence intervals (CIs) from multivariable Cox proportional hazards regression models, to assess the risk of depression or antidepressant use in relation to different DMTs and to assess the risk of DMT discontinuation or MS relapse after DMT initiation in relation to depression or antidepressant use. We analyzed all types of DMTs collectively and stratified the analyses by specific DMT, focusing on interferons, dimethyl fumarate, fingolimod, glatiramer acetate, natalizumab, and rituximab.

In all analyses we used time since DMT start as the underlying timescale. In the first model, we adjusted for sex, country of birth, education, age at DMT start, and, to account for an uneven distribution of DMT treatment across the country, geographical region of treatment. In the second model, to account for the collective effect of psychiatric comorbidity, we further adjusted all associations for history of bipolar disorder, anxiety disorders, and all other mental and behavioral disorders at first DMT start. Finally, to account for the impact of MS severity on the studied associations, in the third model we additionally adjusted for disease duration, DMT line, EDSS, and MSIS-29.

We addressed missing data in the Swedish MS Registry using multiple imputation with the fully conditional specification method and 25 imputations with 30 burn-in iterations. We made separate imputations for each outcome and included in the imputation models all covariates, the specific event, and the Nelson–Aalen estimator of the cumulative hazard substituting the time to event.^16^

As patients change DMTs during the course of the disease, we used a sandwich estimator to account for the fact that patients could belong to multiple treatment cohorts. We considered associations with a two-sided *p* value ⩽ 0.05 statistically significant.

### Sensitivity analyses

We repeated all analyses after further excluding patients with a history of depression longer than 5 years prior to first DMT start (*N* = 184).

Because SSRIs are considered as a first line treatment of depression in current guidelines, we also repeated all analyses after restricting our definition of antidepressants to SSRIs only (ATC code N06AB).

We performed all analyses using Stata software, version 16.1 (StataCorp, College Station, TX). The Regional Ethical Review Board in Stockholm, Sweden, approved this study. Data are available upon approval from the respective register holders.

## Results

Baseline characteristics at the start of first DMT of the 3803 patients included in the analyses are presented in Table 1. The median age at diagnosis of RRMS was 35.3 years (interquartile range (IQR) = 16.3).

**Table 1.** Baseline characteristics at the start of first DMT of patients diagnosed with RRMS in the Swedish MS Registry, January 2010 to September 2018, without a history of depression diagnosis or antidepressant prescription fill in the prior 5 years.

	First DMT, *N* (%)						
	Interferons	Dimethyl fumarate	Fingolimod	Glatiramer acetate	Natalizumab	Rituximab	All^a^
	*N* = 1618	*N* = 579	*N* = 131	*N* = 214	*N* = 442	*N* = 699	*N* = 3803
Female	1113 (68.8)	397 (68.6)	84 (64.1)	162 (75.7)	295 (66.7)	450 (64.4)	2575 (67.7)
Country of birth							
Sweden	1444 (89.2)	508 (87.7)	110 (84.0)	198 (92.5)	394 (89.1)	621 (88.8)	3383 (89.0)
Other	174 (10.8)	70 (12.1)	21 (16.0)	16 (7.5)	48 (10.9)	77 (11.0)	418 (11.0)
Education							
Primary or lower secondary	151 (9.3)	58 (10.0)	15 (11.5)	22 (10.3)	60 (13.6)	70 (10.0)	386 (10.2)
Upper secondary	702 (43.4)	245 (42.3)	59 (45.0)	102 (47.7)	202 (45.7)	302 (43.2)	1668 (43.9)
Postsecondary or postgraduate	754 (46.6)	267 (46.1)	54 (41.2)	88 (41.1)	170 (38.5)	316 (45.2)	1703 (44.8)
Age at DMT start (years)							
9–20	82 (5.1)	30 (5.2)	14 (10.7)	12 (5.6)	51 (11.5)	39 (5.6)	232 (6.1)
21–40	949 (58.6)	376 (64.9)	83 (63.4)	131 (61.2)	323 (73.1)	393 (56.2)	2312 (60.8)
41–60	557 (34.4)	160 (27.6)	33 (25.2)	67 (31.3)	67 (15.2)	250 (35.8)	1,189 (31.3)
61–78	30 (1.9)	13 (2.3)	1 (0.8)	4 (1.9)	1 (0.2)	17 (2.4)	70 (1.8)
Geographical region of treatment							
North Sweden	166 (10.3)	57 (9.8)	12 (9.2)	47 (22.0)	57 (12.9)	176 (25.2)	533 (14.0)
Stockholm	349 (21.6)	131 (22.6)	25 (19.1)	34 (15.9)	73 (16.5)	242 (34.6)	879 (23.1)
Southeast Sweden	240 (14.8)	73 (12.6)	26 (19.9)	21 (9.8)	45 (10.2)	72 (10.3)	492 (12.9)
South Sweden	318 (19.7)	100 (17.3)	33 (25.2)	57 (26.6)	91 (20.6)	56 (8.0)	673 (17.7)
Uppsala-Örebro	243 (15.0)	77 (13.3)	10 (7.6)	29 (13.6)	59 (13.4)	94 (13.5)	529 (13.9)
West Sweden	302 (18.7)	141 (24.4)	25 (19.1)	26 (12.2)	117 (26.5)	59 (8.4)	697 (18.3)
History of clinical diagnoses of mental and behavioral disorders^b^							
Bipolar disorder	3 (0.2)	3 (0.5)	1 (0.8)	1 (0.5)	1 (0.2)	2 (0.3)	11 (0.3)
Anxiety disorders	37 (2.3)	20 (3.5)	1 (0.8)	8 (3.7)	16 (3.6)	25 (3.6)	109 (2.9)
Other^c^	28 (1.7)	22 (3.8)	3 (2.3)	5 (2.3)	26 (5.9)	28 (4.0)	113 (3.0)
MS severity scales, median (IQR)^d^							
EDSS	1.0 (2.0)	1.0 (2.0)	1.5 (2.5)	1.0 (2.0)	2.0 (2.0)	2.0 (1.5)	1.5 (2.5)
MSIS-29 physical	1.3 (0.8)	1.4 (0.8)	1.4 (0.9)	1.5 (1.1)	1.8 (1.5)	1.5 (1.1)	1.4 (0.9)
MSIS-29 psychological	2 (1.2)	2.0 (1.3)	2.2 (1.3)	2.6 (1.3)	2.3 (1.4)	2.2 (1.4)	2.1 (1.5)

DMT: disease-modifying therapies; EDSS: Expanded Disability Status Scale, IQR: interquartile range; MS: multiple sclerosis; MSIS-29: MS Impact Scale; *N* = number of individuals; RRMS: relapsing-remitting MS.

a Including other DMTs (alemtuzumab, daclizumab, hematopoietic stem cell transplantation, mitoxantrone, and teriflunomide).

b Diagnosed within 5 years prior to DMT start.

c All mental and behavioral disorders except depression, bipolar disorder, and anxiety disorders.

d After imputing missing data.

Missing data were <1% for all variables except EDSS (36%–38% of all DMT episodes), and physical and psychological component of MSIS-29 (45%–48% of all DMT episodes).

During follow-up, 43.9% of the patients were ever treated with interferons (*N* = 1667) for a total exposure time of 3779.5 years, whereas 28.3% were ever treated with dimethyl fumarate (*N* = 1077, total exposure time = 1884.2 years), 13.1% with fingolimod (*N* = 497, total exposure time = 1102.7 years), 10.8% with glatiramer acetate (*N* = 411, total exposure time = 848.0 years), 23.0% with natalizumab (*N* = 874, total exposure time = 2015.4 years), 43.2% with rituximab (*N* = 1644, total exposure time = 3195.6 years), and 9.1% with other DMTs (*N* = 347, total exposure time = 594.3 years).

### Depression and antidepressant use in relation to different DMTs

Eleven point five % patients received a first diagnosis of depression or used antidepressants after a median time of 1.59 years from DMT start (IQR = 2.30). The risk of depression or antidepressant use was lower during treatment with rituximab, compared with treatment with interferons (Table 2). Stratifying the analysis on sex showed no clear evidence of effect modification (Supplementary Tables 1–2).

**Table 2.** Associations of DMTs with the risk of depression or antidepressant use among RRMS patients (*N* = 3803).

	Model 1^a^	Model 2^a^	Model 3^a^
	HR (95% CI)	HR (95% CI)	HR (95% CI)
Interferons	Ref.	Ref.	Ref.
Dimethyl fumarate	0.80 (0.60–1.06)	0.79 (0.59–1.05)	0.85 (0.62–1.15)
Fingolimod	0.74 (0.51–1.09)	0.75 (0.51–1.11)	0.75 (0.50–1.14)
Glatiramer acetate	1.11 (0.77–1.61)	1.12 (0.77–1.61)	0.79 (0.52–1.19)
Natalizumab	1.13 (0.88–1.47)	1.12 (0.87–1.45)	0.97 (0.73–1.28)
Rituximab	0.78 (0.61–1.00)	0.77 (0.60–0.99)*	0.72 (0.54–0.96)**

CI: confidence interval; DMTs: disease-modifying therapies; EDSS: Expanded Disability Status Scale; HR: hazard ratio; MS: multiple sclerosis; MSIS-29: MS Impact Scale; N: number of individuals; RRMS: relapsing-remitting MS.

a Model 1: adjusted for sex, country of birth, education, age at DMT start, and geographical region of treatment. Time since DMT start was used as the underlying timescale.

Model 2: further adjusted for history of bipolar disorder, anxiety, and other mental and behavioral disorders in addition to the variables adjusted for in Model 1.

Model 3: further adjusted for disease duration, DMT line, EDSS, and MSIS-29 in addition to the variables adjusted for in Model 2.

* *p* = 0.043; **p = 0.024.

### DMT discontinuation or MS relapse in relation to depression or antidepressant use

The median time from DMT start to DMT discontinuation was 1.81 years (IQR = 2.39). DMT discontinuation was followed by DMT switch for most patients, with 3480 patients (91.5%) still on treatment by the end of follow-up, 291 patients with no recorded DMT after their last DMT interruption (7.6%), and 32 censored (0.8%). The median time from DMT start to MS relapse was 1.73 years (IQR = 2.45). The overall risk of DMT discontinuation after DMT initiation did not differ by depression diagnosis or antidepressant use (Table 3), nor did the overall risk of an MS relapse (Table 4).

**Table 3.** Associations of depression or antidepressant use with the risk of DMT discontinuation among RRMS patients (*N* = 3803), stratified analyses by DMTs.

	Model 1^a^	Model 2^a^	Model 3^a^
	HR (95% CI)	HR (95% CI)	HR (95% CI)
All DMTs			
Depression diagnosis	1.01 (0.85–1.20)	1.01 (0.85–1.20)	1.03 (0.85–1.25)
Antidepressant prescription	1.04 (0.94–1.15)	1.04 (0.95–1.15)	1.06 (0.94–1.19)
Interferons			
Depression diagnosis	1.59 (1.21–2.09)*	1.58 (1.21–2.08)*	1.51 (1.15–1.98)**
Antidepressant prescription	1.29 (1.09–1.54)**	1.29 (1.09–1.53)**	1.14 (0.94–1.37)
Dimethyl fumarate			
Depression diagnosis	0.87 (0.54–1.39)	0.85 (0.53–1.37)	0.75 (0.46–1.21)
Antidepressant prescription	1.08 (0.82–1.43)	1.09 (0.82–1.44)	0.85 (0.63–1.16)
Fingolimod			
Depression diagnosis	1.41 (0.83–2.39)	1.41 (0.82–2.42)	1.34 (0.76–2.37)
Antidepressant prescription	1.51 (1.11–2.07)***	1.54 (1.12–2.12)****	1.47 (1.04–2.08) *****
Glatiramer acetate			
Depression diagnosis	0.70 (0.33–1.47)	0.69 (0.32–1.47)	0.56 (0.26–1.20)
Antidepressant prescription	1.12 (0.80–1.56)	1.10 (0.79–1.53)	0.97 (0.68–1.37)
Natalizumab			
Depression diagnosis	1.10 (0.68–1.80)	1.09 (0.66–1.79)	0.98 (0.60–1.65)
Antidepressant prescription	1.35 (1.02–1.77)******	1.33 (1.01–1.75)*******	1.19 (0.89–1.59)
Rituximab			
Depression diagnosis	1.91 (0.82–4.46)	1.95 (0.85–4.45)	1.57 (0.63–3.91)
Antidepressant prescription	1.17 (0.63–2.15)	1.16 (0.62–2.16)	0.88 (0.45–1.74)

CI: confidence interval; DMTs: disease-modifying therapies; EDSS: Expanded Disability Status Scale; HR: hazard ratio; MS: multiple sclerosis; MSIS-29: MS Impact Scale; *N*: number of individuals; RRMS: relapsing-remitting MS.

a Model 1: adjusted for sex, country of birth, education, age at DMT start, and geographical region of treatment. Time since DMT start was used as the underlying timescale.

Model 2: further adjusted for history of bipolar disorder, anxiety, and other mental and behavioral disorders in addition to the variables adjusted for in Model 1.

Model 3: further adjusted for disease duration, DMT line, EDSS, and MSIS-29 in addition to the variables adjusted for in Model 2.

* *p* = 0.001; **p = 0.003; ***p = 0.009; ****p = 0.007; *****p = 0.030; ******p = 0.031; *******p = 0.038.

**Table 4.** Associations of depression or antidepressant use with the risk of MS relapse among RRMS patients (*N* = 3803), stratified analyses by DMTs.

	Model 1^a^	Model 2^a^	Model 3^a^
	HR (95% CI)	HR (95% CI)	HR (95% CI)
All DMTs			
Depression diagnosis	1.54 (0.89–2.66)	1.57 (0.90–2.74)	1.50 (0.85–2.65)
Antidepressant prescription	1.19 (0.84–1.71)	1.21 (0.84–1.73)	1.21 (0.82–1.79)
Interferons			
Depression diagnosis	1.75 (0.67–4.52)	1.87 (0.73–4.80)	1.65 (0.63–4.35)
Antidepressant prescription	1.29 (0.69–2.42)	1.40 (0.75–2.61)	1.25 (0.64–2.43)
Dimethyl fumarate			
Depression diagnosis	0.59 (0.07–4.53)	0.69 (0.09–5.36)	0.66 (0.08–5.11)
Antidepressant prescription	1.01 (0.35–2.87)	1.08 (0.38–3.09)	1.15 (0.39–3.42)
Fingolimod			
Depression diagnosis	1.93 (0.41–8.91)	1.10 (0.09–13.05)	0.75 (0.06–9.51)
Antidepressant prescription	1.61 (0.64–4.07)	1.48 (0.59–3.73)	0.90 (0.33–2.44)
Glatiramer acetate			
Depression diagnosis	1.22 (0.15–9.72)	1.41 (0.18–11.00)	1.45 (0.17–12.18)
Antidepressant prescription	1.46 (0.48–4.44)	1.70 (0.56–5.15)	1.52 (0.47–4.99)
Natalizumab			
Depression diagnosis	3.61 (1.35–9.61)*	3.13 (1.21–8.09)**	2.60 (0.91–7.41)
Antidepressant prescription	2.02 (0.89–4.61)	2.01 (0.87–4.61)	2.08 (0.84–5.17)
Rituximab			
Depression diagnosis	1.31 (0.31–5.56)	1.39 (0.32–5.99)	1.21 (0.26–5.58)
Antidepressant prescription	0.82 (0.28–2.40)	0.83 (0.28–2.44)	0.78 (0.23–2.63)

CI: confidence interval; DMTs: disease-modifying therapies; EDSS: Expanded Disability Status Scale; HR: hazard ratio; MS: multiple sclerosis; MSIS-29: MS Impact Scale; *N*: number of individuals; RRMS: relapsing-remitting MS.

a Model 1: adjusted for sex, country of birth, education, age at DMT start, and geographical region of treatment. Time since DMT start was used as the underlying timescale.

Model 2: further adjusted for history of bipolar disorder, anxiety, and other mental and behavioral disorders in addition to the variables adjusted for in Model 1.

Model 3: further adjusted for disease duration, DMT line, EDSS, and MSIS-29 in addition to the variables adjusted for in Model 2.

* *p* = 0.010; **p = 0.018.

After stratifying the analyses of treatment discontinuation by DMTs (Table 3), we found a higher risk of interferon discontinuation among patients with depression when compared to patients without depression and a higher risk of fingolimod discontinuation among patients who used antidepressants compared to patients who did not use antidepressants.

### Sensitivity analyses

Further excluding patients with a history of depression longer than 5 years prior DMT start rendered similar results to the main analyses although with wider CIs (Supplementary Tables 3–5), as did restricting the definition of antidepressants to SSRIs only (Table 5).

**Table 5.** Associations of DMTs with the risk of depression or SSRIs use, and of SSRIs use with the risk of DMT discontinuation or MS relapse among RRMS patients (*N* = 3803).

	Model 1^a^	Model 2^a^	Model 3^a^
	HR (95% CI)	HR (95% CI)	HR (95% CI)
Risk of depression or SSRIs use			
Interferons	Ref.	Ref.	Ref.
Dimethyl fumarate	0.82 (0.59–1.13)	0.81 (0.59–1.12)	0.86 (0.61–1.21)
Fingolimod	0.77 (0.51–1.17)	0.79 (0.52–1.20)	0.77 (0.49–1.22)
Glatiramer acetate	1.13 (0.75–1.70)	1.14 (0.76–1.71)	0.85 (0.54–1.32)
Natalizumab	1.26 (0.96–1.66)	1.24 (0.94–1.63)	1.04 (0.77–1.42)
Rituximab	0.78 (0.59–1.03)	0.76 (0.58–1.01)	0.71 (0.51–0.97)
Risk of DMT discontinuation			
SSRIs prescription	0.99 (0.88–1.10)	0.99 (0.88–1.10)	1.00 (0.88–1.13)
Risk of MS relapse			
SSRIs prescription	1.16 (0.77–1.75)	1.18 (0.78–1.78)	1.16 (0.76–1.78)

CI: confidence interval; DMTs: disease-modifying therapies; EDSS: Expanded Disability Status Scale; HR: hazard ratio; MS: multiple sclerosis; MSIS-29: MS Impact Scale; *N*: number of individuals; RRMS: relapsing-remitting MS; SSRIs: selective serotonin reuptake inhibitors.

a Model 1: adjusted for sex, country of birth, education, age at DMT start, and geographical region of treatment. Time since DMT start was used as the underlying timescale.

Model 2: further adjusted for history of bipolar disorder, anxiety, and other mental and behavioral disorders in addition to the variables adjusted for in Model 1.

Model 3: further adjusted for disease duration, DMT line, EDSS, and MSIS-29 in addition to the variables adjusted for in Model 2.

## Discussion

Using a nationwide population-based cohort of RRMS patients, we found that patients treated with rituximab had a lower risk of receiving a depression diagnosis or using antidepressants as compared with patients treated with interferons. We also found that patients with a depression diagnosis had an increased risk of discontinuing interferons compared with patients without depression, while patients who used antidepressants had an increased risk of discontinuing fingolimod treatment compared with patients who did not use antidepressants. Being diagnosed with depression or using antidepressants did not influence the overall risk of DMT discontinuation or MS relapse.

Fingolimod and natalizumab have been reported to improve depressive symptoms in MS patients with pre-existing depressive symptoms.^17–20^ On the contrary, other DMTs have been associated with worsening of depressive symptoms, in particular among patients on interferon beta-1b treatment with a past history of depression.^21^ Early trials also indicated a higher frequency of depressive symptoms, relative to placebo, in patients treated with interferon beta-1b.^22^ These associations, though, seemed to be explained by history of psychiatric disorders prior to initiation of interferon beta-1b and were not replicated by larger subsequent studies.^22–24^ Still, depression is listed as a possible side effect not only for interferon beta-1b, but all currently approved DMTs.^25^

Although rituximab is not an approved treatment for RRMS it has become the most widely used DMT in Sweden based on a series of studies in real world cohorts demonstrating superior clinical outcomes than regular MS DMTs.^26^ It is therefore of interest that we detected a lower risk of depression or antidepressant use in relation to use of rituximab, as compared with interferons, and that the risk reduction was of greater numerical magnitude than with dimethyl fumarate, fingolimod, glatiramer acetate, and natalizumab. Together with the fact that patients on rituximab show a significantly higher drug persistence than patients with comparative DMTs,^26–28^ this result indicates a better effectiveness and tolerability profile of rituximab. A more speculative hypothesis is that this observation may be related to depression-related inflammatory pathways.^5^ Natalizumab, and to some degree also dimethyl fumarate and fingolimod, are also considered among highly effective MS DMTs. The absence of a consistently lower risk of depression with these treatments could indicate a role for B cells in relation to depressive symptoms.^29^ However, a causal relationship between B-cell depleting therapies and the risk of depression remains to be shown. It is also important to consider the mode of administration of the studied DMTs when interpreting the results. Rituximab is dosed twice a year. Natalizumab requires monthly visits at an infusion clinic. Dimethyl fumarate and fingolimod are taken orally daily, whereas interferons and glatiramer acetate are given as regular self-administered injections. It is possible that these differences influence the risk of being diagnosed with depression due to varying level of contact with healthcare providers. Further studies are therefore needed to address whether there is a true protective effect of rituximab on the risk of depression or whether the difference compared to interferons is due to a negative effect of the latter.

Additional studies using treatment satisfaction questionnaires and general quality of life scales may shed light on the relation between treatment tolerability and mode of administration and psychological wellbeing. It may be speculated that treatment of depression may bring additional benefits apart from just symptomatic relief by modulating MS-associated inflammatory responses and clinical outcomes. However, currently there is not sufficient data to support the general use of antidepressants among MS patients according to the American Academy of Neurology Guidelines^30^ and the consequences of untreated psychiatric comorbidity on the general wellbeing and quality of life of MS patients remains unknown.^31^

In contrast to earlier studies reporting a negative effect of depression on DMT adherence^32^ and MS relapse,^33^ we did not find a significant effect of depression or antidepressant use on the overall risk of DMT discontinuation or experiencing an MS relapse. In the analysis of specific DMTs, we detected a higher risk of interferon discontinuation in patients diagnosed with depression. Our finding is in accord with the previously suggested association between a fatigue-depression interaction factor and interferon beta-1b discontinuation using an open label trial of 72 MS patients.^34^ We speculate that our result might partially be explained by the lack of effectiveness of interferons, which was the main reason for interferon discontinuation among depressed patients. However, it remains to be shown whether the reported lack of effectiveness of interferon treatment was based on objective signs of ongoing inflammatory disease activity or a possible interaction with depressive symptoms perceived by the patient.

In the analysis of specific DMTs we also detected a higher risk of fingolimod discontinuation in patients treated with antidepressants; however, this association must be interpreted with caution given the chance of random findings.

We also noted a higher risk of MS relapses among patients on natalizumab with depression compared with non-depressed patients. While this observation was confounded by MS severity, it may still indicate that this patient group might be more prone to psychogenic relapses. Although psychogenic causes are well-described as a differential diagnosis to MS,^35^ this is more rarely investigated in patients with a confirmed MS diagnosis.

There are certain limitations with this study. First, we had access to psychiatric diagnoses from specialized inpatient and outpatient care but not from primary care, with the consequence of not being able to capture the less complicated depression cases usually treated within the primary care. However, the fact that we had access to complete data of filled prescriptions in pharmacies from both specialized and primary healthcare/general practitioners enabled us to assess all pharmacological interventions, even if this then may include also other indications for these drug classes such as anxiety disorders. Second, we were not able to account for resolution of depression and we did not have access to information on non-pharmacological interventions for depression such as psychological therapy. We also did not have access to family history of depression and were, therefore, unable to investigate whether heritability of depression would modify the association of DMT groups with depression. Third, although the access to more precise disease phenotype information from the Swedish MS Registry made it possible to correct for additional disease-related factors that differed between the DMT groups, we did not account for lesion load and relapse activity, hence residual confounding cannot be ruled out. Finally, although our estimate of a protective effect of rituximab on depression was statistically significant, the CIs were relatively wide, and results should be replicated in larger samples. Larger samples would also give the possibility to separately investigate whether the psychiatric vulnerability of patients with preexisting depression prior to DMT start is also modified by DMT initiation.

In sum, using a contemporary nationwide incident cohort of RRMS patients we detected a lower risk of depression or antidepressant use in patients treated with rituximab, compared with patients treated with interferons. Occurrence of depression or antidepressant use did not affect the overall risk of DMT discontinuation or MS relapse. Additional studies will be needed to explore the existence of a possible causal link and whether biological and/or psychological factors contribute to the observed differences, or whether interferons increase the risk of depression.

## Supplemental Material

sj-docx-1-msj-10.1177_13524585211031128 – Supplemental material for Risk of depression in multiple sclerosis across disease-modifying therapies Click here for additional data file. Supplemental material, sj-docx-1-msj-10.1177_13524585211031128 for Risk of depression in multiple sclerosis across disease-modifying therapies by Elisa Longinetti, Thomas Frisell, Simon Englund, Johan Reutfors, Fang Fang and Fredrik Piehl in Multiple Sclerosis Journal

sj-docx-2-msj-10.1177_13524585211031128 – Supplemental material for Risk of depression in multiple sclerosis across disease-modifying therapies Click here for additional data file. Supplemental material, sj-docx-2-msj-10.1177_13524585211031128 for Risk of depression in multiple sclerosis across disease-modifying therapies by Elisa Longinetti, Thomas Frisell, Simon Englund, Johan Reutfors, Fang Fang and Fredrik Piehl in Multiple Sclerosis Journal

sj-docx-3-msj-10.1177_13524585211031128 – Supplemental material for Risk of depression in multiple sclerosis across disease-modifying therapies Click here for additional data file. Supplemental material, sj-docx-3-msj-10.1177_13524585211031128 for Risk of depression in multiple sclerosis across disease-modifying therapies by Elisa Longinetti, Thomas Frisell, Simon Englund, Johan Reutfors, Fang Fang and Fredrik Piehl in Multiple Sclerosis Journal

sj-docx-4-msj-10.1177_13524585211031128 – Supplemental material for Risk of depression in multiple sclerosis across disease-modifying therapies Click here for additional data file. Supplemental material, sj-docx-4-msj-10.1177_13524585211031128 for Risk of depression in multiple sclerosis across disease-modifying therapies by Elisa Longinetti, Thomas Frisell, Simon Englund, Johan Reutfors, Fang Fang and Fredrik Piehl in Multiple Sclerosis Journal

sj-docx-5-msj-10.1177_13524585211031128 – Supplemental material for Risk of depression in multiple sclerosis across disease-modifying therapies Click here for additional data file. Supplemental material, sj-docx-5-msj-10.1177_13524585211031128 for Risk of depression in multiple sclerosis across disease-modifying therapies by Elisa Longinetti, Thomas Frisell, Simon Englund, Johan Reutfors, Fang Fang and Fredrik Piehl in Multiple Sclerosis Journal
