# Supplementary material for: Risk of depression in multiple sclerosis across disease-modifying therapies
Source: Mult Scler. 2021 Jul 15;28(4):632–41. doi: 10.1177/13524585211031128 (PMC8961249; doi:10.1177/13524585211031128)
Supplement: sj-docx-1-msj-10.1177_13524585211031128 – Supplemental material for Risk of depression in multiple sclerosis across disease-modifying therapies [file sj-docx-1-msj-10.1177_13524585211031128.docx]

| **Supplementary Table 1.** Associations of DMTs with the risk of depression or antidepressant prescription fill among male RRMS patients (N=1,228). | | | |
| --- | --- | --- | --- |
|  | **Model 1** ^a^ | **Model 2** ^a^ | **Model 3** ^a^ |
|  | HR (95% CI) | HR (95% CI) | HR (95% CI) |
| Interferons | Ref. | Ref. | Ref. |
| Dimethyl fumarate | 0.68 (0.37-1.22) | 0.67 (0.37-1.21) | 0.77 (0.41-1.44) |
| Fingolimod | 0.98 (0.52-1.85) | 0.99 (0.53-1.88) | 1.07 (0.56-2.03) |
| Glatiramer acetate | 1.06 (0.49-2.27) | 1.02 (0.47-2.21) | 0.71 (0.31-1.64) |
| Natalizumab | 1.35 (0.81-2.24) | 1.32 (0.80-2.20) | 1.23 (0.71-2.15) |
| Rituximab | 0.81 (0.50-1.32) | 0.80 (0.49-1.29) | 0.75 (0.44-1.30) |
| ^a^ Model 1: adjusted for country of birth, education, age at DMT start, and geographical region of treatment. Time since DMT start was used as the underlying time scale.  Model 2: further adjusted for history of bipolar disorder, anxiety, and other mental and behavioral disorders in addition to the variables adjusted for in Model 1.  Model 3: further adjusted for disease duration, DMT line, EDSS, and MSIS-29 in addition to the variables adjusted for in Model 2.  Abbreviations: CI=confidence interval; DMT=disease modulatory therapies; EDSS=expanded disability status scale; HR=hazard ratio; MS=multiple sclerosis; MSIS-29=MS impact scale; N=number of individuals; RRMS=relapsing-remitting MS. | | | |
