# Supplementary material for: Risk of depression in multiple sclerosis across disease-modifying therapies
Source: Mult Scler. 2021 Jul 15;28(4):632–41. doi: 10.1177/13524585211031128 (PMC8961249; doi:10.1177/13524585211031128)
Supplement: sj-docx-2-msj-10.1177_13524585211031128 – Supplemental material for Risk of depression in multiple sclerosis across disease-modifying therapies [file sj-docx-2-msj-10.1177_13524585211031128.docx]

| **Supplementary Table 2.** Associations of DMTs with the risk of depression or antidepressant prescription fill among female RRMS patients (N=2,575). | | | |
| --- | --- | --- | --- |
|  | **Model 1** ^a^ | **Model 2** ^a^ | **Model 3** ^a^ |
|  | HR (95% CI) | HR (95% CI) | HR (95% CI) |
| Interferons | Ref. | Ref. | Ref. |
| Dimethyl fumarate | 0.84 (0.61-1.17) | 0.83 (0.60-1.16) | 0.87 (0.61-1.24) |
| Fingolimod | 0.64 (0.40-1.04) | 0.66 (0.40-1.07) | 0.62 (0.36-1.07) |
| Glatiramer acetate | 1.13 (0.75-1.72) | 1.15 (0.75-1.74) | 0.82 (0.51-1.31) |
| Natalizumab | 1.07 (0.79-1.44) | 1.06 (0.78-1.43) | 0.88 (0.63-1.23) |
| Rituximab | 0.77 (0.58-1.04) | 0.77 (0.57-1.02) | **0.70 (0.50-0.98)** |
| ^a^ Model 1: adjusted for country of birth, education, age at DMT start, and geographical region of treatment. Time since DMT start was used as the underlying time scale.  Model 2: further adjusted for history of bipolar disorder, anxiety, and other mental and behavioral disorders in addition to the variables adjusted for in Model 1.  Model 3: further adjusted for disease duration, DMT line, EDSS, and MSIS-29 in addition to the variables adjusted for in Model 2.  Abbreviations: CI=confidence interval; DMT=disease modulatory therapies; EDSS=expanded disability status scale; HR=hazard ratio; MS=multiple sclerosis; MSIS-29=MS impact scale; N=number of individuals; RRMS=relapsing-remitting MS. | | | |
