# Supplementary material for: Risk of depression in multiple sclerosis across disease-modifying therapies
Source: Mult Scler. 2021 Jul 15;28(4):632–41. doi: 10.1177/13524585211031128 (PMC8961249; doi:10.1177/13524585211031128)
Supplement: sj-docx-5-msj-10.1177_13524585211031128 – Supplemental material for Risk of depression in multiple sclerosis across disease-modifying therapies [file sj-docx-5-msj-10.1177_13524585211031128.docx]

| **Supplementary Table 5**. Associations of depression or antidepressants prescription fill with the risk of MS relapse among RRMS patients after further excluding patients with history of depression longer than five years prior first DMT start (N=3,619), stratified analyses by DMTs. | | | |
| --- | --- | --- | --- |
|  | **Model 1** ^a^ | **Model 2** ^a^ | **Model 3** ^a^ |
|  | HR (95% CI) | HR (95% CI) | HR (95% CI) |
| **All DMTs** | | | |
| Depression diagnosis | N/A | N/A | N/A |
| Antidepressant prescription | 1.11 (0.73-1.70) | 1.11 (0.73-1.70) | 1.18 (0.75-1.87) |
| **Interferons** | | | |
| Depression diagnosis | N/A | N/A | N/A |
| Antidepressant prescription | 1.30 (0.65-2.62) | 1.40 (0.70-2.79) | 1.35 (0.63-2.86) |
| **Dimethyl fumarate** | | | |
| Depression diagnosis | N/A | N/A | N/A |
| Antidepressant prescription | 0.91 (0.28-2.94) | 0.96 (0.30-3.14) | 1.08 (0.31-3.70) |
| **Fingolimod** | | | |
| Depression diagnosis | N/A | N/A | N/A |
| Antidepressant prescription | 1.93 (0.61-6.11) | 1.47 (0.37-5.90) | 0.88 (0.20-3.76) |
| **Glatiramer acetate** | | | |
| Depression diagnosis | N/A | N/A | N/A |
| Antidepressant prescription | 1.49 (0.43-5.20) | 1.63 (0.46-5.73) | 1.48 (0.37-5.82) |
| **Natalizumab** | | | |
| Depression diagnosis | N/A | N/A | N/A |
| Antidepressant prescription | 1.56 (0.54-4.54) | 1.56 (0.53-4.62) | 1.96 (0.59-6.47) |
| **Rituximab** | | | |
| Depression diagnosis | N/A | N/A | N/A |
| Antidepressant prescription | 0.52 (0.12-2.24) | 0.52 (0.12-2.26) | 0.50 (0.10-2.53) |
| ^a^ Model 1: adjusted for sex, country of birth, education, age at DMT start, and geographical region of treatment. Time since DMT start was used as the underlying time scale.  Model 2: further adjusted for history of bipolar disorder, anxiety, and other mental and behavioral disorders in addition to the variables adjusted for in Model 1.  Model 3: further adjusted for disease duration, DMT line, EDSS, and MSIS-29 in addition to the variables adjusted for in Model 2.  Abbreviations: CI=confidence interval; DMT=disease modulatory therapies; EDSS=expanded disability status scale; HR=hazard ratio; MS=multiple sclerosis; MSIS-29=MS impact scale; N=number of individuals; N/A=not applicable due to low number of depression episodes; RRMS=relapsing-remitting MS. | | | |
